# Changes in Carbohydrate Composition in Fermented Total Mixed Ration and Its Effects on *in vitro* Methane Production and Microbiome

**DOI:** 10.3389/fmicb.2021.738334

**Published:** 2021-11-05

**Authors:** Yang Li, Jingyi Lv, Jihong Wang, Shuang Zhou, Guangning Zhang, Bingdong Wei, Yukun Sun, Yaxue Lan, Xiujing Dou, Yonggen Zhang

**Affiliations:** ^1^College of Animal Sciences and Technology, Northeast Agriculture University, Harbin, China; ^2^Jilin Academy of Agricultural Sciences, Changchun, China

**Keywords:** fermented total mixed ration, carbohydrate component, methane yield, ruminal fermentation, rumen microbiome

## Abstract

The purpose of this experiment was to investigate the changes of carbohydrate composition in fermented total mixed diet and its effects on rumen fermentation, methane production, and rumen microbiome *in vitro.* The concentrate-to-forage ratio of the total mixed ration (TMR) was 4:6, and TMR was ensiled with lactic acid bacteria and fibrolytic enzymes. The results showed that different TMRs had different carbohydrate compositions and subfractions, fermentation characteristics, and bacterial community diversity. After fermentation, the fermented total mixed ration (FTMR) group had lower contents of neutral detergent fiber, acid detergent fiber, starch, non-fibrous carbohydrates, and carbohydrates. In addition, lactic acid content and relative abundance of *Lactobacillus* in the FTMR group were higher. Compared with the TMR group, the *in vitro* ammonia nitrogen and total volatile fatty acid concentrations and the molar proportion of propionate and butyrate were increased in the FTMR group. However, the ruminal pH, molar proportion of acetate, and methane production were significantly decreased in the FTMR group. Notably, we found that the relative abundance of ruminal bacteria was higher in FTMR than in TMR samples, including *Prevotella*, *Coprococcus*, and *Oscillospira*. At the same time, we found that the diversity of methanogens in the FTMR group was lower than that in the TMR group. The relative abundance of *Methanobrevibacter* significantly decreased, while the relative abundances of *Methanoplanus* and *vadinCA11* increased. The relative abundances of *Entodinium* and *Pichia* significantly decreased in the FTMR group compared with the TMR group. These results suggest that FTMR can be used as an environmentally cleaner technology in animal farming due to its ability to improve ruminal fermentation, modulate the rumen microbiome, and reduce methane emissions.

## Introduction

Methane is a greenhouse gas with an abundance second only to carbon dioxide, and the greenhouse efficiency is 25 times that of the equivalent amount of carbon dioxide (IPCC, 2007), which can enhance radiative forcing and greenhouse effects, and further aggravate disasters such as climate deterioration, global warming, and land desertification. Methanogenesis is a fundamental rumen metabolic process; it is also the main cause of nutrient loss and energy waste in feed (Takahashi et al., 1997; Takahashi, 2001) and contributes to 11–17% of the global greenhouse gas emissions (Beauchemin et al., 2009; Goel and Makkar, 2012). Therefore, whether from the perspective of environmental protection or animal production, it is extremely necessary to reduce methane emissions from ruminants.

Methane reduction is an important research topic not only in the field of ruminant nutrition but also in environmental protection. In animal production, methane emissions can be reduced through dietary manipulation and feed additives. However, the ideal methane inhibitor needs to have many characteristics, such as being extremely specific and long-lasting, safe for use in animals, and not leaving behind residues in edible products (Van Nevel and Demeyer, 1996). In addition, ionophore antibiotics (monensin) have been banned in the production of heifers and dairy cows in some countries. Therefore, it is a safer and more effective method to reduce methane production by using ruminant diet nutrition control methods.

Fermented total mixed ration (FTMR) is a new type of ruminant feeding technology, in which the finished total mixed rations (TMR) are tightly wrapped by a professional baler and a special plastic stretch film, and the TMR is sealed by a specific single or compound strain (Liu et al., 2016a,b) for anaerobic fermentation so that the TMR can be stored for a certain time. Our previous studies proved that FTMR could improve the feed efficiency and lactation performance of dairy cows (Zhang et al., 2020b); we also discovered that the contents of structural carbohydrates (NDF and ADF) in FTMR were significantly reduced with an increased lactic acid content (Zhang et al., 2020a,b). Diet composition can have a major effect on methane production (Benchaar et al., 2001). Highly concentrated diets should reduce methane production (Granja-Salcedo et al., 2016). The type of carbohydrate in rumen fermentation regulates methane production mainly by affecting the rumen pH value and the total amount of rumen microbiome. Reducing the proportion of structural carbohydrates in the diet reduces rumen pH and thus reduces methane production by inhibiting the activity of methanogens (Russell, 1998). Changes in fermentation substrates in TMR also affect rumen fermentation types and volatile fatty acid synthesis pathways (Cao et al., 2010). The inhibition of methane production is normally accompanied by increased in propionate production (Wolin, 1975), which uses hydrogen and lactic acid. Furthermore, the relative abundance of rumen bacteria and protozoa, especially methanogens, is an important reason to explain the variation in ruminal methane production. It is important to fully understand the microbial changes involved in rumen methane production to clarify the mechanism by which FTMR regulates it. Therefore, we hypothesized that FTMR might decrease methane production by altering the rumen fermentation pattern and inhibiting the major methanogens or bacteria and protozoa related to methanogenesis in the rumen.

So the current study aimed to evaluate the effects of fermentation on the carbohydrate composition and fermentation index of TMR and investigate the effects of FTMR on *in vitro* ruminal fermentation, methane production, and rumen microbiome. The results from this study are expected to improve the carbohydrate composition of TMR concomitant with decreased CH_4_ production for cleaner environmental animal agriculture.

## Materials and Methods

### Preparation of Total Mixed Ration

Total mixed ration in this study was prepared using compound feed in the Ruminant Nutrition Laboratory from Northeast Agriculture University. Corn silage, alfalfa hay, steam-flaked corn, soybean meal, rapeseed meal, dry corn gluten feed, distillers dried grains with soluble (DDGS), and premix were procured from the Songhuajiang Dairy Farm (Harbin city, Heilongjiang province, China). The ingredient and chemical composition are shown in Table 1. The TMR was formulated with a forage-to-concentrate ratio of 60:40 (DM basis) and met the animals’ requirements for nutrition based on the Cornell-Penn-Miner dairy model (Version 3.0.10; Cornell University, Ithaca, NY; University of Pennsylvania, Kennett Square, PA; and William H. Miner Agricultural Research Institute, Chazy, NY). Alfalfa hay was chopped to a length of 3–4 cm.

**TABLE 1:** Ingredient and nutrient composition of the experimental ration (DM basis).

**Composition**	**Ingredient,%**
Corn silage	42.76
Alfalfa hay	17.27
Steam-flaked corn	18.32
Soybean meal	7.78
Rapeseed meal	5.94
Dry corn gluten feed	2.90
DDGS	2.92
Premix^a^	2.11
**Nutrient level,%**	
NE_L_ (Mcal/kg of DM)^b^	1.56
CP	16.39
NDF	41.24
ADF	27.52
Ash	8.12
Starch	20.26
EE	3.45
NFC^c^	30.44

*^a^The premix contained (on a DM basis): 99.17% ash, 14.25% Ca, 5.40% P, 4.93% Mg, 0.05% K, 10.64% Na, 2.95% Cl, 0.37% S, 12 mg/kg Co, 500 mg/kg Cu, 4,858 mg/kg Fe, 25 mg/kg I, 800 mg/kg Mn, 10 mg/kg Se, 1,800 mg/kg Zn, 180,000 IU/kg vitamin A, 55,000 IU/kg vitamin D and 1,500 IU/kg vitamin E.*

*^b^Calculated based on the (Ministry of Agriculture of P. R. China [MOA], 2004).*

*^c^NFC = OM—(CP + NDF + fat).*

The TMR was ensiled with a combination of lactic acid bacteria [LAB; *Lactobacillus plantarum* CGMCC10516 and *Lactobacillus buchneri* BNCC189797, applied at a ratio of 1:1; theoretical final application rate of 10^6^ colony-forming units (cfu)/g of fresh matter (FM)] and fibrolytic enzyme (1 g fibrolytic enzyme per 1 kg FM [EN; 10,000 U/g activity, XS Biotechnology Co., Ltd., Beijing, China)]. Additives were homogenously mixed into TMR using a hand sprayer. After proper mixing, the TMR was packed into a 20 × 30 cm plastic laboratory fermentation bag (Chuangjia Packaging Material Co., Ltd., Wenzhou, China). The air was subsequently removed by a vacuum packing machine (Maige Automation Equipment Co., Ltd., Qingdao, China). Eight bags (1 ensiling day × 1 treatment × 8 replicates) were prepared and incubated indoor at ambient temperature (20–30°C) for 30 days. Three bags (3 replicates) of non-fermented total mixed and FTMR were opened to analyze them for fermentation characteristics, chemical compositions, and *in vitro* fermentation. Five samples (5 replicates) per two treatments were analyzed to determine the microbial community.

### Fermentation Index, Carbohydrate Composition, and Subfractions of Carbohydrates Using Cornell Net Carbohydrate and Protein System Analysis

After 30 days, 50 g of the wet ensiled silage samples of FTMR and the non-fermented TMR were homogenized with 200 mL of sterilized distilled water and stored at 4°C overnight (Cai et al., 1999). The pH was measured using a pH meter (Sartorius basic pH meter, Göttingen, Germany). The concentrations of lactic acid (LA), acetic acid (AA), and butyric acid (BA) were measured by high-performance liquid chromatography (Yuan et al., 2016), while ammonia nitrogen (NH_3_-N) was determined according to the phenol/hypochlorite method (Broderick and Kang, 1980).

Fermented or non-fermented TMR were dried at 60°C for 48 h and ground with a high-speed universal mill to pass through a 1-mm sieve grind. The dry matter (DM, AOAC 930.15) content of fermented or non-fermented TMR samples was determined by the methods of AOAC (2005). The contents of neutral detergent fiber (NDF), acid detergent fiber (ADF), acid detergent lignin (ADL), and residual ash were measured according to the filtration method (Van Soest et al., 1991) using an Ankom 220 fiber analyzer (Ankom Technology Corp., Macedon, NY, United States), and α-amylase and sodium sulfite were used for the NDF procedure. Starch content was determined using the Megazyme Total Starch Assay Procedure (product no: K-TSTA; Megazyme International Ireland Ltd., Wicklow, Ireland). All analyses were conducted in triplicate. The lignin content was calculated as ADL—residual ash, non-fibrous carbohydrate (NFC) as OM—CP—NDF—fat; and carbohydrate (CHO) as 100—EE—CP—ash. The carbohydrate subfractions were partitioned into CA, CB_1_, CB_2_, and CC according to the Cornell Net Carbohydrate and Protein System (CNCPS) (Sniffen et al., 1992).

### *In vitro* Incubation

The *in vitro* incubation procedures were as previously described (Li et al., 2019). The substrates were prepared with fermented or non-fermented TMR by drying and grinding through a 0.45 mm sieve. Rumen fluid was collected via rumen cannula before the morning feeding from three steers of the yellow cattle breed, filtered with four-layer cheesecloth, and mixed with preheated artificial saliva (Menke and Steingass, 1988) at a ratio of 2:1 (buffer: ruminal fluid, v:v). The ruminal fluid (150 mL) that was buffered was dispensed into prewarmed 200-mL incubation flasks. Two grams of each substrate was blended with the buffered ruminal fluid in each incubation flask, which was incubated at 39°C for 24 h in water. The methane production was measured by a real-time *in vitro* fermentation system (produced by Jilin Academy of Agricultural Sciences, code Qtfxy-6), which was tested for the effluent gas discharged from each incubation flask. Nitrogen (purity 99.99%) was passed into the incubation flask from the bottom at a speed of 200 mL/min. Methane was carried by nitrogen into an AGM10 sensor (Sensors Europe GmbH, Erkrath, FRG), and the concentration of methane was measured and recorded every 6 min (Sun et al., 2017). After determining methane production, the pH of the culture liquor was determined using a pH meter (Sartorius basic pH meter, Göttingen, Germany). For analysis of NH_3_-N and volatile fatty acids (VFAs), 1 ml of 25% meta-phosphoric acid was added to 5 ml of culture liquor and stored at −20°C until analysis. The VFA concentration was determined by gas chromatography as previously described (Stewart and Duncan, 1985). Ammonia nitrogen was determined according to the phenol/hypochlorite method (Broderick and Kang, 1980). The microbial crude protein (MCP) was determined following the procedures of Hu et al. (2005). All analyses were conducted in triplicate.

### Microbial Community Diversity Analysis

The remaining silage subsample (10 g) from fermented or non-fermented TMR was mixed with 40 mL of saline solution (NaCl, 0.90 g/g) and shaken at 120 r/m for 2 h. The filtered liquor through gauze was centrifuged at 10,000 r/m for 10 min at 4°C. The supernatant was discarded, but the deposit was suspended in 3 mL of saline solution (Zhang et al., 2020a). This procedure was repeated for good precipitation. According to the manufacturer’s protocol, genomic DNA from fermented or non-fermented TMR was extracted using Fast DNA SPIN extraction kits (MP Biomedicals, Santa Ana, CA, United States). Metagenomic DNA was extracted from ruminal samples using the cetyltrimethylammonium bromide (CTAB) method but with bead beating (Wang et al., 2019). The quality of the DNA extracts was evaluated using agarose (1%) electrophoresis, while the DNA concentration was determined using the Qubit dsDNA BR Assay Kit (Invitrogen Corporation, United States) on a Qubit 2.0 fluorometer (Invitrogen Corporation, United States). The extracted DNA was subjected to PCR amplification in triplicate using the Accuprime Taq DNA Polymerase System (Invitrogen, Carlsbad, CA, United States). For bacterial analysis, the V3-V4 region of the 16S rRNA gene was amplified using primers 338F (5′-ACTCCTRCGGGAGGCAGCAG-3′) and 806R (5′-GGACTACCVGGGTATCTAAT-3′) (Mao et al., 2015). For ciliate protozoal analysis, V3-V4 and signature regions 1–2 of the 18S rRNA gene were amplified using primers PSSU-316F (5′-GCTTTCGWTGGTAGTGTATT-3′) (Sylvester et al., 2004) and GIC758R (5′-CAACTGTCTCTATKAAYCG-3′) (Ishaq and Wright, 2014). For archaeal analysis, the V3-V4 region of the 16S rRNA gene was amplified using primers F341 (5′-CTACGGGGYGCASCAG-3′) (Wei et al., 2013) and R806 (5′-GGACTACVVGGGTATCTAATC-3′). After purification and quantification of the PCR products, the DNA fragments of the community were sequenced by double-terminal (paired-end) sequencing using an Illumina MiSeq platform (Wuhan Frasergen Bioinformatics Co., Ltd., Wuhan, China). Sequencing data were processed using the Quantitative Insights Into Microbial Ecology (QIIME, v1.8.0)^1^ pipeline as previously described (Caporaso et al., 2010). Alpha diversity measurements, including Chao1 richness estimates, Shannon and Simpson diversity indices, and observed species, were calculated for each sample. The microbiome was compared as beta diversity using the distance matrices generated from weighted UniFrac and principal coordinates analysis (PCoA). Taxa abundance at the genus level was statistically compared between the samples for fermented or non-fermented TMR and their abundance of rumen microbiome.

### Statistical Analyses

Data were analyzed using SAS software (version 9.4, SAS Institute Inc., Cary, NC). Data for rumen microbiome were subjected to the non-parametric Wilcoxon Two-Sample Test to examine the relative abundance of the genus-level bacterial community in TMR or FTMR and the effect of TMR after fermentation. The other data were analyzed using the one-way ANOVA procedure. Significance was declared at *P* < 0.05 and trends at 0.05 ≤ *P* < 0.10. Standard errors of the mean and standard deviation (rumen microbiome) are reported.

## Results and Discussion

Compared with the control group, the FTMR group had lower DM (*P* = 0.0009), NDF (*P* = 0.006), ADF (*P* = 0.001), starch (*P* = 0.0002), NFC (*P* < 0.0001), CHO (*P* = 0.0006) contents, and CB_1_ (*P* = 0.0003) and CB_2_ (*P* = 0.03) subfractions. Moreover, the fractions of CA_1_ (*P* = 0.002) and CC (*P* = 0.01) in TMR increased significantly after fermentation (Table 2). This indicates that fermentation improves the availability of carbohydrates in TMR. It can be seen from the results of this experiment that the changes in carbohydrate composition of TMR by cofermentation of lactic acid bacteria and cellulose enzymes are obvious, which also lay a foundation for the change in rumen fermentation type and the reduction in methane production. A high-concentrate diet should decrease methane emissions (Granja-Salcedo et al., 2016); therefore, a diet with a higher proportion of roughage was designed in this study to emphasize the role of FTMR in decreasing methane emissions. The low DM content in fermentation TMR may be due to the decomposition of nutrients into liquids and gases by microorganisms (Kim et al., 2016). Cao et al. (2010) found that the contents of DM, NFC, NDF, and ADF in FTMR were lower than those in TMR, which was consistent with the results of this study. The contents of starch, NDF, and ADF in FTMR were low, which might be caused by lactic acid bacteria hydrolyzing digestible carbohydrates in the feed to produce lactic acid. In addition, the degradation of plant cell walls by cellulase to increase the content of water-soluble carbohydrates could also be a major reason for the decrease in NDF and ADF (Liu et al., 2016a). The digestible carbohydrate components in TMR will be used as substrates for lactic acid production to facilitate the fermentation of the diet (Xing et al., 2009). The carbohydrate components of CNCPS could reflect the degradability of dietary carbohydrates in the rumen. The increase in the CA fraction and decrease in the fractions of CB_1_ and CB_2_ in FTMR are related to the conversion of starch and digestible fiber in feed to lactate by lactobacillus. The lower content of NDF and ADF in the FTMR diet may be due to the fact that lactic acid bacteria hydrolyzed more digestible plant cells to produce acid during silage (Liu et al., 2016a). Moreover, inoculated LAB have higher fermentation efficiency than epiphytic LAB to transform sugars into lactic acid (Yuan et al., 2015). In this study, a fibrolytic enzyme was added to TMR to degrade the plant cell wall and produce lactate as a substrate (Xing et al., 2009); however, DM loss due to fermentation increased the CC fraction.

**TABLE 2:** Chemical profiles and carbohydrate subfractions in fermented or non-fermented TMR.

**Items^a^**	**FTMR**	**TMR**	**SEM**	***P*-value**
**chemical composition**				
DM (%)	42.63	47.26	0.37	0.0009
NDF (%/DM)	37.48	41.24	0.49	0.006
ADF (%/DM)	22.52	27.52	0.44	0.001
Lignin (%/DM)	10.26	10.84	0.29	0.22
Starch (%/DM)	14.47	20.26	0.31	0.0002
NFC (%/DM)	28.12	30.44	0.089	< 0.0001
CHO (%/DM)	65.60	71.67	0.44	0.0006
**subfractions of carbohydrates (using the CNCPS)**				
CA (%/CHO)	26.40	19.80	0.61	0.002
CB_1_ (%/CHO)	22.06	28.26	0.38	0.0003
CB_2_ (%/CHO)	14.01	15.63	0.33	0.03
CC (%/CHO)	37.53	36.31	0.19	0.01

*^a^DM, dry matter; NDF, neutral detergent fiber; ADF, acid detergent fiber; NFC, non-fibrous carbohydrate; CHO, carbohydrate. CA is the rapidly degradable carbohydrate subfraction; CB_1_ is the intermediately degradable carbohydrate subfraction; CB_2_ is the slowly degradable carbohydrate subfraction; CC is the fraction of CHO that is considered to be undegradable.*

As shown in Table 3, fermentation reduced the pH value of TMR to below 4.2. Compared with TMR, ammonia nitrogen, lactic, and acetic acid concentration in FTMR was significantly increased. Butyric acid was detected in both TMR groups, indicating that the quality of both TMR groups was good. The rapid decline in pH depends on lactic acid produced by lactic acid bacteria and cellulases degrading carbohydrates, which is important in reducing nutrient loss caused by the growth of undesirable microorganisms during the early stage of fermentation (Liu et al., 2016a). When the pH value of silage is lower than 4.2, the quality of silage can be guaranteed (Wen et al., 2017). Large amounts of starch and fermentable carbohydrates in TMR provide sufficient fermentation substrates for lactic acid bacteria and cellulase, leading to a significant increase in lactic acid content in FTMR. Previous studies have found that LAB containing *Lactobacillus plantarum* and *Lactobacillus buchneri* inoculated in TMR produced a large amount of lactic and acetic acid during fermentation (Zhang et al., 2020b). Liu et al. (2016a) also found that the addition of microbial preparations and enzyme preparations in FTMR could increase the fermentation of lactic acid bacteria. With the extension of fermentation time, lactic acid is converted into acetic acid, which is consistent with the results of Alli et al. (2010). Although the pH of TMR was significantly decreased, we did not find any negative effects of FTMR on the performance of dairy cows in previous experiments (Zhang et al., 2020b). The ammonia-nitrogen content in FTMR was significantly increased, which proved that there was more decomposition of true protein in the feed after fermentation (Xing et al., 2009), but the increase in non-protein nitrogen content also provided an abundant nitrogen source for the rumen.

**TABLE 3:** Fermentation characteristics of fermented or non-fermented TMR.

**Items**	**FTMR**	**TMR**	**SEM**	***P*-value**
pH	3.96	5.86	0.018	0.0001
NH_3_-N	0.94	0.68	0.002	<0.0001
Lactic acid	6.80	5.20	0.16	0.002
Acetic acid	2.99	2.15	0.030	<0.0001
Butyric acid	ND	ND		

**FIGURE 1:**
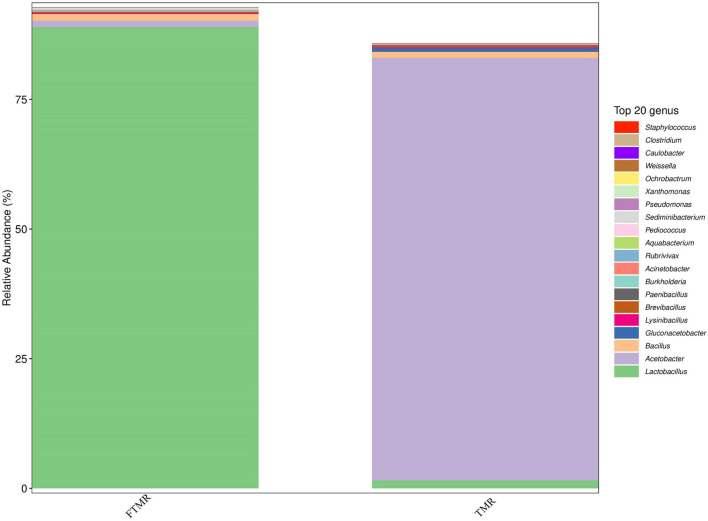
Relative abundance of bacterial under genera level in fermented or non-fermented TMR.

**FIGURE 2:**
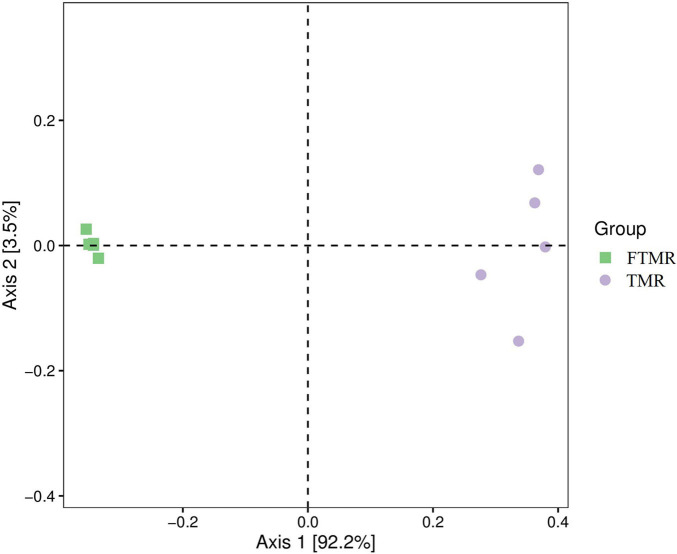
Principal Co-ordinates analysis of the bacterial community in fermented or non-fermented TMR.

Table 4 and Figure 1 show that lactic acid bacteria became the absolute dominant bacterial genus in the TMR after fermentation (*P* < 0.0001). The PCoA plots (Figure 2) clearly indicate the microbial community variability and show that the distance between the FTMR and the TMR was far. *Lactobacillus* and *Acetobacter* were the two main bacterial genera that changed after fermentation. In this experiment, the addition of exogenous *Lactobacillus* increased the relative abundance of lactic acid bacteria in the TMR. Moreover, the addition of cellulase degraded the plant cell wall in the diet and increased the relative abundance of *Lactobacillus* (Xing et al., 2009). In the non-fermented TMR, it may be that the *Acetobacter* was attached to the raw materials of the TMR, making *Lactobacillus* the dominant bacteria in mixing and stirring. In conclusion, *Lactobacillus* and cellulase catalyze the fermentation process, promote the growth of *Lactobacillus*, and increased the content of lactic acid and acetic acid, thereby reducing the pH value (pH < 4.2), inhibiting the growth of other bacteria (*Acetobacter*, *Gluconacetobacter*, and *Paenibacillus*), and making *Lactobacillus* the dominant bacteria.

**TABLE 4:** Relative abundance (%) of 10 most predominant genera in fermented or non-fermented TMR.

**Items**	**FTMR**	**TMR**	***P*-value**
*Lactobacillus*	88.99 ± 6.47	1.52 ± 0.84	**
*Acetobacter*	1.21 ± 0.72	81.52 ± 8.78	**
*Bacillus*	1.25 ± 0.61	1.13 ± 0.75	NS
*Gluconacetobacter*	0.0074 ± 0.014	0.68 ± 0.16	**
*Lysinibacillus*	0.20 ± 0.12	0.17 ± 0.22	NS
*Brevibacillus*	0.20 ± 0.14	0.17 ± 0.17	NS
*Paenibacillus*	0.043 ± 0.037	0.28 ± 0.31	*
*Burkholderia*	0.15 ± 0.19	0.0019 ± 0.0019	**
*Acinetobacter*	0.048 ± 0.056	0.075 ± 0.049	NS
*Rubrivivax*	0.12 ± 0.16	0.0015 ± 0.0024	**

**P < 0.01, *P < 0.05, NS, not significant.

**FIGURE 3:**
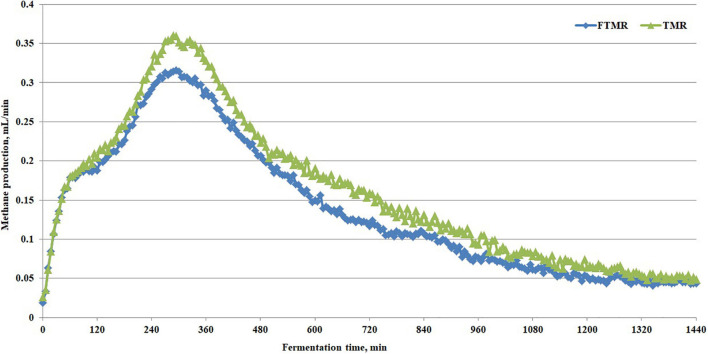
Methane production curve *in vitro* with fermented or non-fermented TMR.

In the *in vitro* gas production test, we further found that FTMR increased ammonia nitrogen (*P* = 0.003), total volatile fatty acid concentration (*P* = 0.0048), and molar proportion of propionate (*P* = 0.03) and butyrate (*P* = 0.001) and decreased rumen pH (*P* = 0.03), molar proportion of acetate (*P* = 0.006), acetate to propionate ratio (*P* = 0.01) and methane production (*P* = 0.0007) (Table 5). Figure 3 shows the real-time methane production *in vitro* with fermented or non-fermented TMR. The higher non-protein nitrogen content in FTMR may lead to an increase in ammonia-nitrogen content in the rumen, and this result was consistent with that of Cao et al. (2010). In this experiment, it was found that the content of NDF and ADF in TMR decreased, and the acetic acid yield decreased after fermentation. More structural carbohydrates in FTMR are converted into rapidly degradable carbohydrates. However, the increase in the CA fraction in FTMR may increase volatile fatty acid concentration and a decrease in pH *in vitro*. There was a negative correlation between rumen pH and fermentation gas production (Waghorn, 1991). Reducing the proportion of structural carbohydrates in the diet decreases rumen pH and thus reduces methane production by inhibiting the activity of methanogens (Russell, 1998). Bacteria degraded the feed concentrate and CA in TMR to produce propionic acid, which decreased rumen pH and the acetate to propionate ratio. At the same time, lactic acid from FTMR and rumen fermentation may have further increased the amount of propionic acid using the hydrogen produced by the fermentation reaction (Cao et al., 2010). The inhibition of methane production by FTMR may be due to indirect or direct inhibition (or both) of methanogens via a decline in H_2_ production due to reduced acetate and more propionate production (Cieslak et al., 2013; Kumar et al., 2014). In addition, the high content of lactic acid in FTMR is also key to regulating methane production. Propionic acid was produced when lactic acid in the feed was secondarily fermented in the rumen by lactate-utilizing bacteria (*Megasphaera elsdenii*, *Selenomonas ruminantium*, and *Veillonella parvula*) (Dawson et al., 1997; Russell and Wallace, 1997). Propionic acid fermentation uses electrons to reduce methane production. Moreover, lactic acid in the rumen uses hydrogen for conversion to propionic acid, and the reduction of hydrogen also inhibits the formation of methane from hydrogen and carbon dioxide (Moss et al., 2000). In addition, shifting ruminal fermentation to more propionate would inhibit hydrogen-producing bacteria (*Ruminococcus albus*, *Ruminococcus flavefaciens*, and protozoa) (Cobellis et al., 2016), so it is necessary to understand further the effect of FTMR on the rumen microbiome *in vitro*.

**TABLE 5:** *In vitro* pH, NH_3_-N, MCP, VFA, and methane production of fermented or non-fermented TMR.

**Items**	**FTMR**	**TMR**	**SEM**	***P*-value**
Ph	6.73	6.84	0.024	0.03
NH_3_-N (mg/dL)	10.73	8.29	0.27	0.003
Total VFA^a^ (mmol/L)	61.37	54.05	0.92	0.0048
**Molar proportion, mmol/100 mmol**				
Acetate	66.61	70.57	0.54	0.006
Propionate	22.26	19.91	0.48	0.03
Butyrate	11.13	9.52	0.14	0.001
Acetate/Propionate	3.00	3.55	0.088	0.01
MCP^b^ (mg/mL)	128.26	121.07	2.43	0.10
Methane (mL)	199.47	231.13	2.33	0.0007

*^a^Total VFA, total volatile fatty acid.*

*^b^MCP, microbial crude protein.*

As seen in Table 6, FTMR improved the Chao1 (*P* = 0.001) and observed species (*P* = 0.002) of rumen bacteria and tended to reduce the Shannon index of methanogens (*P* = 0.07), which proved that FTMR had higher ruminal bacterial abundance and lower methanogen diversity. In addition, we found that FTMR significantly increased the relative abundance of *Prevotella* (*P* < 0.05), *Coprococcus* (*P* < 0.05) and *Oscillospira* (*P* < 0.05); and significantly reduced the relative abundance of *Entodinium* (*P* < 0.05) and *Pichia* (*P* < 0.05). Although it increased the relative abundance of *Methanoplanus* (*P* < 0.05) and *vadinCA11* (*P* < 0.05), it had a significant reduction effect on *Methanobrevibacter* (*P* < 0.05) (Table 7). In an experiment to regulate methane production, bacterial genera were analyzed to understand the effects of exogenous substances on the rumen microbiome (Martínez-Fernández et al., 2014; Zhou et al., 2017, 2020). In the present study, 16S rRNA sequencing was used to comparatively examine the influence of FTMR on ruminal bacteria, protozoa, and methanogens. *Prevotella*, a gram-negative genus of *Bacteroidetes*, increased with FTMR supplementation, possibly due to increased fermentation substrates such as non-protein nitrogen and reduced competition from other bacteria. It has been suggested that FTMR could increase ruminal protein degradation and ammonia concentrations (Zhang et al., 2020b). We also observed that FTMR significantly increased ammonia nitrogen, which is in agreement with a previous study (Cao et al., 2010). Studies have shown that *Coprococcus* is involved in an important metabolic pathway in the rumen (Martinez-Fernandez et al., 2017). In cows with higher feeding efficiency, the abundance of genes related to the acrylate pathway has increased, which involves the conversion of lactic acid to propionic acid (Martinez-Fernandez et al., 2017). In general, the main pathway for propionic acid synthesis in highly efficient animals is the acrylate pathway rather than the succinate pathway (Shabat et al., 2016). Shabat et al. (2016) found that the relative abundance of *Coprococcus* in the rumen of cows with low CH_4_ emissions and high feed efficiency was higher, consistent with the present experimental results. Furthermore, Mackie et al. (2003) found that the abundance of *Oscillospira*, which is the bacterium involved in the degradation of the plant cell wall (Yanagita et al., 2003), in the rumen was diet-dependent, and that *Oscillospira* may play an important role in producing or utilizing amino acids (Hua et al., 2017). Therefore, we think that the change in the relative abundance of *Oscillospira* was related to the change in ammonia nitrogen and structural carbohydrate content in FTMR. The number of rumen protozoa is closely related to the number of methanogens and methane production, and *Entodinium* had the highest contribution rate to methane production (Ranilla et al., 2007). Eugène et al. (2004) conducted a meta-analysis of data in the published literature and found that defaunation could increase the concentration of propionic acid, and the increase in propionic acid production would reduce methane production through the utilization of hydrogen. Moreover, almost all rumen ciliates have methanogens on the surface. It has also been found that there are endosymbiotic methanogens in the cytosol of rumen ciliates, and the number of methanogens in the cytosol is far greater than the number of methanogens bound to the surface of the ciliates (Finlay et al., 1994). Thirty-seven percent of methane in ruminants is produced by protozoan-related methane bacteria (Finlay et al., 1994). In this experiment, the relative abundance of *Entodinium* and *Pichia* decreased, and the rumen methanogens decreased, thereby reducing rumen methane production. Although changes in the abundance of rumen bacteria and protozoa have a positive impact on *in vitro* methane production, studies have indicated that ruminal methane production may be much more influenced by the relative abundance of archaea rather than by the microbial population structure (Wallace, 2004; Duarte et al., 2017). *Methanobacterium*, *Methanobrevibacter*, *Methanomicrobium*, and *Methanosarcine* are four methanogens widely existing in the rumen of ruminants, among which *Methanobrevibacter* is the most dominant genus (Jarvis et al., 2000). In this study, FTMR significantly reduced the relative abundance of *Methanobrevibacter* in the rumen, which may be due to the reduction in the abundance of protozoa, resulting in the lack of attachments, which in turn inhibited the activity of methanogens and reduced methane production. It is also possible that FTMR decreased the rumen pH and inhibited the activity of methanogens in the rumen, thus reducing methane production. Interestingly, we found that FTMR increased the relative abundance of *Methanoplanus* and *Vadinca11*, which may be caused by the decrease in the abundance of other methanogens. However, the causes of the increase in these genera are not well understood, and further studies are needed to elucidate their mechanism.

**TABLE 6:** Changes in richness and diversity of rumen microbiome in fermenters fed fermented or non-fermented TMR.

**Item**	**FTMR**	**TMR**	**SEM**	***P*-value**
**Bacteria**				
Chao1	5818.79	4689.70	101.54	0.001
Observed species	5214.67	4107.93	107.31	0.002
Shannon	9.55	9.11	0.19	0.17
Simpson	0.013	0.018	0.0027	0.24
**Protozoa**				
Chao1	1147.92	1161.54	299.01	0.98
Observed species	1139.5	1155.3	300.38	0.97
Shannon	7.33	6.78	0.98	0.71
Simpson	0.057	0.094	0.048	0.62
**Methanogens**				
Chao1	625.26	650.09	30.70	0.59
Observed species	605.23	629.93	28.45	0.57
Shannon	5.43	6.20	0.22	0.07
Simpson	0.101	0.056	0.021	0.20

**TABLE 7:** Relative abundance (%) of 10 most predominant genera of rumen microbiome in fermenters fed fermented or non-fermented TMR.

**Rumen microbiome**	**FTMR**	**TMR**	***P*-value**
**Bacteria**			
*Pseudobutyrivibrio*	13.31 ± 2.18	22.98 ± 7.23	NS
*Ruminobacter*	19.08 ± 1.26	9.51 ± 14.18	NS
*Prevotella*	12.36 ± 2.01	8.14 ± 1.21	*
*Butyrivibrio*	2.62 ± 0.45	6.11 ± 3.01	NS
*Anaeroplasma*	1.83 ± 0.16	4.48 ± 2.33	NS
*Coprococcus*	2.37 ± 0.34	1.29 ± 0.061	*
*Ruminococcus*	1.22 ± 0.13	1.33 ± 0.44	NS
*Succiniclasticum*	1.34 ± 0.17	1.08 ± 0.34	NS
*RFN20*	1.18 ± 0.27	1.00 ± 0.13	NS
*Oscillospira*	1.04 ± 0.13	0.60 ± 0.029	*
**Protozoa**			
*Dasytricha*	17.49 ± 29.79	34.04 ± 28.23	NS
*Entodinium*	2.61 ± 2.34	7.73 ± 0.45	*
*Isotricha*	0.31 ± 0.54	3.66 ± 3.07	NS
*Metadinium*	0.22 ± 0.20	1.81 ± 1.59	NS
*Abrus*	1.38 ± 2.37	0.006 ± 0.011	NS
*Ophryoscolex*	0.081 ± 0.14	0.87 ± 0.78	NS
*Pseudoentodinium*	0.20 ± 0.34	0.61 ± 0.97	NS
*Tetratrichomonas*	0.24 ± 0.42	0.56 ± 0.15	NS
*Pichia*	0.00 ± 0.00	0.48 ± 0.24	*
*Diploplastron*	0.00 ± 0.00	0.43 ± 0.39	NS
**Methanogens**			
*Methanobrevibacter*	55.18 ± 12.96	88.38 ± 3.13	*
*Methanoplanus*	33.26 ± 12.35	3.73 ± 2.24	*
*vadinCA11*	4.27 ± 0.94	1.39 ± 0.48	*
*Methanosphaera*	1.16 ± 0.45	1.41 ± 0.43	NS
*Methanimicrococcus*	0.35 ± 0.25	0.60 ± 0.63	NS
*Candidatus_Nitrososphaera*	0.020 ± 0.035	0.0089 ± 0.015	NS
*Methanosarcina*	0.017 ± 0.029	0.00 ± 0.00	NS
*Methanobacterium*	0.0011 ± 0.0019	0.0044 ± 0.0077	NS
*Methanosaeta*	0.0022 ± 0.0019	0.0011 ± 0.0019	NS
*Thermococcus*	0.0022 ± 0.0039	0.00 ± 0.00	NS

*P < 0.05. NS, not significant.

## Conclusion

Compared to non-fermented TMR, FTMR has a lower structural carbohydrate content and a higher CA fraction, lactic acid content and relative abundance of *Lactobacillus*. FTMR increases the molar proportion of propionate and butyrate, decreases ruminal pH and the ratio of acetate to propionate and lowers methane emissions. The effect of FTMR on reducing methane emissions seems to result from the conversion of lactic acid to propionic acid and the decrease in protozoa and methanogens in the rumen. FTMR could be used as an environmentally cleaner technology in animal farming due to its ability to improve ruminal fermentation of diets and reduce methane production.

## Data Availability Statement

The raw data supporting the conclusions of this article will be made available by the authors, without undue reservation.

## Ethics Statement

The animal study was reviewed and approved by all animal studies were conducted according to the animal care and use guidelines of the Animal Care and Use Committee of Animal Science and Technology College, Northeast Agricultural University.

## Author Contributions

YLi, XD, and YZ designed the experiment. JL, JW, and SZ conducted the experiment and collected samples. YS and YLa analyzed the samples and data. YLi wrote the manuscript. YLi, JL, GZ, and BW revised the manuscript. All authors contributed to the article and approved the submitted version.

## Conflict of Interest

The authors declare that the research was conducted in the absence of any commercial or financial relationships that could be construed as a potential conflict of interest.

## Publisher’s Note

All claims expressed in this article are solely those of the authors and do not necessarily represent those of their affiliated organizations, or those of the publisher, the editors and the reviewers. Any product that may be evaluated in this article, or claim that may be made by its manufacturer, is not guaranteed or endorsed by the publisher.

## Footnotes

1
http://qiime.org/

## Abbreviations

AA: acetic acid
ADL: acid detergent lignin
ADF: acid detergent fiber
BA: butyric acid
CH_4_: methane
cfu: colony-forming unit
CP: crude protein
CHO: carbohydrate
CNCPS: Cornell net carbohydrate and protein system
DM: dry matter
DNA: deoxyribonucleic acid
DDGS: dried distiller grains with solubles
EE: ether extract
EN: enzyme
FM: fresh matter
FTMR: fermented total mixed ration
GLM: general linear model
LA: lactic acid
LAB: lactic acid bacteria
NDF: neutral detergent fiber
NFC: non-fibrous carbohydrate
NH_3_-N: ammonia nitrogen
OM: organic matter
PCoA: principal coordinates analysis
TMR: total mixed ration
VFA: volatile fatty acid.
